# Adjunctive systemic corticosteroids in pediatric orbital cellulitis: a systematic review and meta-analysis

**DOI:** 10.3389/fped.2026.1794826

**Published:** 2026-04-20

**Authors:** Emily S. Acker, Gabriela Martin Gonzalez, Dylan Davie, Ratna B. Basak

**Affiliations:** 1 College of Medicine, SUNY Downstate Health Sciences University, Brooklyn, NY, United States; 2 Department of Pediatrics, NYC Health+Hospitals/Kings County, Brooklyn, NY, United States

**Keywords:** corticosteroids, length of stay, meta-analysis, orbital cellulitis, pediatrics, surgical intervention

## Abstract

**Background:**

Orbital cellulitis is a serious pediatric infection that can cause vision- and life-threatening complications. Adjunctive systemic corticosteroids are sometimes added to antibiotics to reduce inflammation, but their effectiveness and safety in children remain uncertain.

**Methods:**

We conducted a PRISMA-compliant systematic review and meta-analysis of studies evaluating adjunctive systemic corticosteroids in children (<21 years) hospitalized with orbital cellulitis. Eligible studies compared corticosteroid plus antibiotics vs. antibiotics alone and reported hospital length of stay (LOS), surgical intervention, pediatric intensive care unit (PICU) admission, or 30-day readmission. Randomized and observational studies were included. Data were pooled using random- or fixed-effects models depending on heterogeneity.

**Results:**

Six studies (*n* = 11,803; 1,811 corticosteroid, 9,992 control) met inclusion criteria. Corticosteroid use was not associated with a statistically or clinically meaningful reduction in LOS (mean difference −0.43 days, 95% CI: −2.34 to 1.49). Corticosteroid use was associated with higher risks of surgery (RR 2.08, 95% CI: 1.47–2.96), PICU admission (RR 1.82, 95% CI: 1.36–2.45), and 30-day readmission (RR 2.53, 95% CI: 1.94–3.29). Certainty of evidence was low to moderate, largely due to observational data.

**Conclusions:**

Adjunctive corticosteroids were not associated with shorter hospitalization and may be associated with increased risks of adverse outcomes. These findings should be interpreted with caution given the observational nature of most included studies and potential confounding. High-quality pediatric randomized controlled trials are needed to better define the role of corticosteroids in orbital cellulitis.

**Systematic Review Registration:**

https://www.crd.york.ac.uk/PROSPERO/view/CRD420251102999, PROSPERO CRD420251102999

## 1 Introduction

Orbital cellulitis is a critical infection of the post-septal tissues, most frequently seen in children as a complication of sinusitis. Without prompt and appropriate treatment, it can quickly escalate to severe complications, such as vision loss, intracranial abscess, or cavernous sinus thrombosis. Management typically includes intravenous antibiotics and, in some cases, surgical drainage; however, adjunctive treatment strategies remain variable.

Systemic corticosteroids are sometimes used as adjunctive therapy due to their anti-inflammatory effects. By reducing orbital inflammation, edema, and intra-orbital pressure, corticosteroids may theoretically accelerate symptom resolution and improve clinical outcomes, such as reducing hospital length of stay. However, their use remains controversial, as immunosuppressive effects may worsen infection, mask clinical progression, or delay recognition of complications (1).

Existing studies evaluating corticosteroid use in orbital cellulitis have reported conflicting results. Smaller studies have suggested potential benefits, including shorter hospitalization and faster symptom improvement (2–4), whereas larger retrospective analyses have demonstrated no clear benefit and, in some cases, an increased risk of adverse outcomes such as surgical intervention, PICU admission, and readmission (1, 5).

Prior systematic reviews have included limited pediatric data and often combined adult and pediatric populations, reducing applicability to younger patients. Notably, the only randomized controlled trial in this area excluded children under 10 years of age, further limiting generalizability to the broader pediatric population (6, 7).

Given these limitations, there remains a need for a comprehensive synthesis of pediatric-specific evidence evaluating the safety and effectiveness of adjunctive corticosteroids in orbital cellulitis.

This systematic review and meta-analysis aims to evaluate the association between adjunctive systemic corticosteroid use and clinical outcomes in children hospitalized with orbital cellulitis.

## 2 Methods

### 2.1 Protocol and registration

This review followed the PRISMA (Preferred Reporting Items for Systematic Reviews and Meta-Analyses) guidelines and adhered to the Cochrane Handbook for Systematic Reviews of Interventions (8, 9). The protocol was registered prospectively in PROSPERO (registration ID: CRD420251102999).

### 2.2 Eligibility criteria

#### 2.2.1 Inclusion criteria

We included studies that focused on pediatric patients under the age of 21 who were hospitalized with a diagnosis of orbital cellulitis, with or without a subperiosteal abscess. We defined the pediatric population as individuals under 21 years of age, consistent with prior pediatric and adolescent medicine studies that include older adolescents within pediatric cohorts. To be eligible, studies needed to evaluate the use of systemic corticosteroids, administered at any dose, route, or timing, as an adjunct to standard antibiotic therapy. The comparison group had to receive standard antibiotic treatment alone, without the use of corticosteroids during the initial phase of care. Studies were required to report at least one of the following outcomes: hospital length of stay, surgical intervention, admission to a pediatric intensive care unit (PICU), or hospital readmission within 30 days. Both randomized controlled trials and observational studies that included a comparator group were considered for inclusion.

#### 2.2.2 Exclusion criteria

We excluded studies that combined adults and children without clearly stratifying results by age, those involving only topical or intranasal corticosteroids, and studies focusing on periorbital cellulitis without clear differentiation from orbital cellulitis. Case reports or case series with fewer than 10 patients were also excluded, as were studies that did not stratify outcomes based on corticosteroid use. Articles not published in English were excluded from this review.

### 2.3 Search strategy

We conducted a comprehensive search of PubMed, Embase, and the Cochrane Library from database inception through June 1st, 2025. Search terms included combinations of: “orbital cellulitis,” “corticosteroids,” “steroids,” “glucocorticoids,” “pediatric,” “adolescent,” and “child,” using Boolean operators (AND, OR). An English language filter was applied. The full search strategy for each database is provided in the Supplementary Material. After removal of duplicates, titles and abstracts were screened for relevance, followed by full-text review based on predefined eligibility criteria.

### 2.4 Study selection

We retrieved studies from electronic databases and used EndNote to remove duplicate entries (10). The remaining records were then uploaded into Rayyan for screening (11). Two reviewers independently assessed each title and abstract for potential inclusion. Full-text articles were reviewed when eligibility remained unclear or met initial criteria. Any disagreements were resolved through discussion, with a third reviewer consulted when needed.

### 2.5 Data extraction

Two reviewers independently extracted data into a standardized form. Extracted variables included study characteristics (design, country, setting, year), patient demographics and clinical features (age, severity, presence of abscess), intervention details (steroid agent, route, dose, duration, timing), and outcomes (hospital length of stay, PICU admission, surgical intervention, 30-day readmission).

### 2.6 Quality assessment

The quality of included non-randomized studies was assessed using the Newcastle–Ottawa Scale (NOS), a validated tool for evaluating observational study quality (12). Studies were categorized as high quality (scores 7–9), fair quality (scores 5–6), or poor quality (scores <5). Two reviewers independently evaluated each study, and discrepancies were resolved through discussion or consultation with a third reviewer.

### 2.7 Statistical analysis

A pairwise meta-analysis was performed using Review Manager (RevMan), version 5.4 (13). Data were collected as mean ± standard deviation (SD) for continuous outcomes, or as event counts and totals for binary outcomes, for the steroid and no-steroid (control) groups. For continuous outcomes such as hospital length of stay (LOS), we calculated pooled mean differences (MDs) with corresponding 95% confidence intervals (CIs). For binary outcomes, including surgical intervention, PICU admission, and 30-day readmission, we calculated pooled risk ratios (RRs).

We used fixed- or random-effects models based on heterogeneity. Heterogeneity was assessed using the I^2^ statistic and Chi-squared test, with I^2^ values of <25% considered low, 25%–50% moderate, and >50% substantial heterogeneity. A random-effects model was used for outcomes with substantial heterogeneity, while a fixed-effects model was applied when heterogeneity was low or moderate (8). Due to inconsistent reporting of adjusted effect estimates and covariate selection across studies, pooled analyses were conducted using unadjusted data to maintain comparability across studies. Adjusted results, when available, were summarized narratively. Reporting bias was assessed using visual inspection of funnel plots for outcomes with a sufficient number of studies (22).

For studies that did not report LOS as mean ± SD, we estimated these values using standard methods. Medians and interquartile ranges were converted using the Meta-Analysis Accelerator, which applies the validated (15) approach assuming approximate normality of the underlying data (14, 15). For studies reporting only ranges or graphical LOS distributions, we used empirical formulas or extracted midpoint values to approximate means and SDs. All transformations were consistent with published methods in prior meta-analyses. To assess the impact of these assumptions, we performed a sensitivity analysis limited to studies reporting mean and SD directly, which yielded similar results.

We evaluated the certainty of evidence for each outcome using the GRADE (Grading of Recommendations, Assessment, Development, and Evaluation) approach. Because all included studies were observational, outcomes were initially rated as low certainty. Certainty was downgraded based on predefined domains, including risk of bias (e.g., lack of adjustment for confounding), inconsistency (e.g., substantial heterogeneity across studies), indirectness, imprecision (e.g., wide confidence intervals), and potential publication bias. Where appropriate, certainty was upgraded for large effect sizes (e.g., RR > 2) or when confounding was likely to reduce rather than exaggerate the observed association (23). GRADE assessments for each outcome are summarized in Table 2, which serves as a summary of findings table.

## 3 Results

### 3.1 Database search

**Figure 1:**
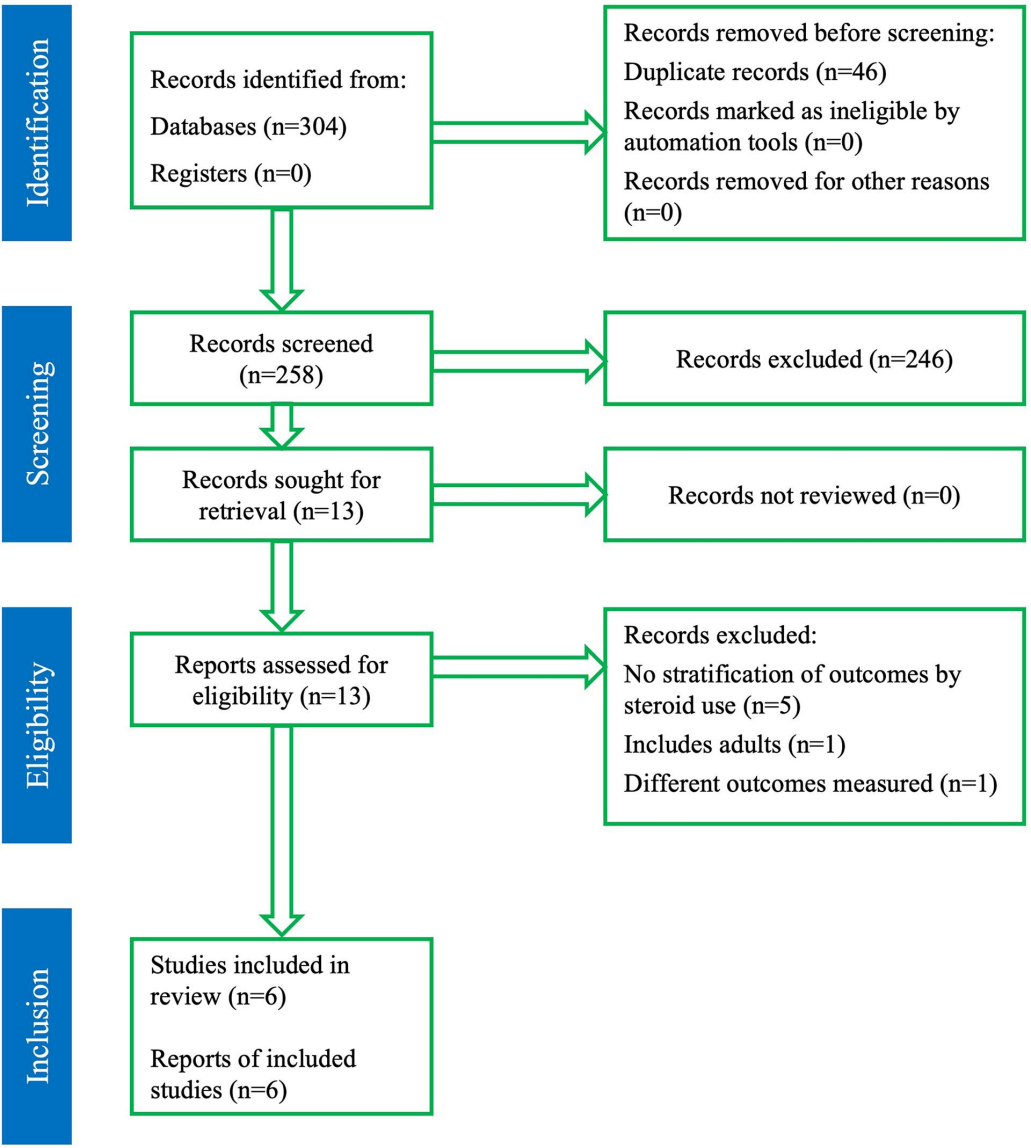
PRISMA flow diagram. Database searches yielded 304 records; 258 unique citations were screened after duplicate removal. Thirteen full-text articles were reviewed, and 6 studies met inclusion criteria for the meta-analysis.

The initial literature search yielded 304 records. After removing 46 duplicates, 258 unique citations were screened by title and abstract. Thirteen articles were selected for full-text review based on relevance to the research question. Of these, six studies met the predefined inclusion criteria and were included in the final systematic review and meta-analysis (1, 3–5, 16, 17). A detailed overview of the study selection process is presented in the PRISMA flow diagram (Figure 1).

### 3.2 Baseline characteristics

This meta-analysis included two prospective and four retrospective cohort studies that met predefined eligibility criteria following a systematic database search and screening protocol. Each study assessed outcomes between a cohort of pediatric patients who received adjunctive systemic corticosteroids and a group who received antibiotics alone, without corticosteroids as part of their initial management. The studies were published between 2005 and 2022 and included a total of 1,811 patients treated with corticosteroids and 9,992 controls. All studies were conducted in the United States and focused on patients under the age of 21 years.

There were different definitions of corticosteroid use across studies, including variations in the type, dose, timing of initiation, and duration of use. In most cases, steroids were started as part of the initial management. However, one study looked specifically at steroids started after the patient had begun to improve clinically, setting it apart from the others (4). The comparator groups were also not defined consistently across studies. Most studies defined control groups as patients who never received steroid therapy. On the other hand, Gill et al. specifically classified controls as patients who did not receive steroids on day 0, regardless of subsequent steroid administration to isolate the effect of early corticosteroid use (5).

Additionally, differences existed in the reporting of outcomes. For example, (1) was the only study that explicitly separated early and late surgical procedures, as well as early and late PICU admissions (1). A summary of key demographic and clinical characteristics across included studies is provided in Table 1.

**Table 1:** Study characteristics: adjunctive corticosteroid use in pediatric orbital cellulitis.

Study ID	Anosike	Chen	Davies	Gill	Leszcynska	Yen
Year	2022	2018	2015	2022	2021	2005
Study Design	Retrospective cohort study	Prospective comparative interventional study	Prospective comparative interventional study	Retrospective cohort study	Retrospective cohort study	Retrospective cohort study
Country	U.S.	U.S.	U.S.	U.S.	U.S.	U.S.
Sample Size	220	43	31	5,832	5,645	23
Age	Mean: 8.3 yrs. Range: 2 mos.—21 yrs.	Mean: 8.9 yrs., Range 5–17 yrs.	Mean: ∼7.9 yrs.	Median: 5.0 yrs. (IQR: 2.0–9.0 yrs.)	Median: 6 yrs. (IQR 3–10 yrs.)	Range:11–15 yrs.
% Male	61%	44%	81%	61%	64.50%	Not specified
Inclusion Criteria	Children ≤ 21 yrs. with orbital cellulitis ± subperiosteal orbital abscess (excluded those with immunodeficiency, malignancy, recent trauma, or recent facial/orbital surgery)	Children <18 yrs. old hospitalized with orbital cellulitis (excluded immunocompromised, CNS involvement, steroid contraindication)	Children aged 1–18 yrs. hospitalized with orbital cellulitis (excluded patients diagnosed with orbital inflammatory syndrome, presenting with intracranial abscess/infection, immunocompromised patients, patients with contraindication to systemic steroids)	Children 2 mos.–18 yrs. with primary diagnosis of orbital cellulitis (excluded those with complex comorbidities, other indications for steroids, and patients who underwent surgery on day 0)	Children <18 yrs. old hospitalized with orbital cellulitis (excluded those with complex chronic conditions and other diagnoses associated with steroid use)	Pediatric patients with subperiosteal abscess due to orbital cellulitis
Corticosteroid Regimen	Systemic corticosteroids. Regimen not specified.	IV dexamethasone 0.3 mg/kg every 6 h. for 3 days. Initiated on admission.	Oral prednisone 1 mg/kg daily for 7 days, initiated when CRP normalized (CRP < 4 mg/dL)	Corticosteroids on day 0, mostly dexamethasone, methylprednisolone, prednisolone	Systemic corticosteroids (88.8% dexamethasone), within first 2 days of admission	IV corticosteroids on admission; regimen not standardized
Comparator group	Antibiotics alone	Antibiotics alone	Antibiotics alone	No corticosteroids on day 0	Antibiotics alone	Antibiotics alone
Outcomes	Hospital LOS, surgical intervention, 30-day readmission	Hospital LOS, surgical intervention	Hospital LOS, surgical intervention, 30-day readmission	Hospital LOS, surgical intervention, PICU admission, 30-day readmission	Hospital LOS, surgical intervention, PICU admission, 30-day readmission	Hospital LOS, surgical intervention
NOS Score	9	8	8	9	9	8

CNS, central nervous system; CRP, C-reactive protein; LOS, length of stay; PICU, pediatric intensive care unit.

### 3.3 Quality assessment

The quality of the six included studies was evaluated using the Newcastle–Ottawa Scale (NOS), assessing studies based on three domains: selection of participants, comparability between groups, and outcome assessment. All studies were rated as high quality, with NOS scores ranging from 8 to 9 out of 9. Four studies received full scores, reflecting strong performance across all domains (1, 5, 16, 17). The remaining two studies scored 8, primarily due to limitations in how they accounted for differences between groups (3, 4). Although all studies were rated as high quality based on NOS scores, residual confounding and bias related to observational study design may still be present. A summary of individual study scores is provided in Table 1.

### 3.4 Outcomes

#### 3.4.1 Hospital length of stay (LOS)

**Figure 2:**
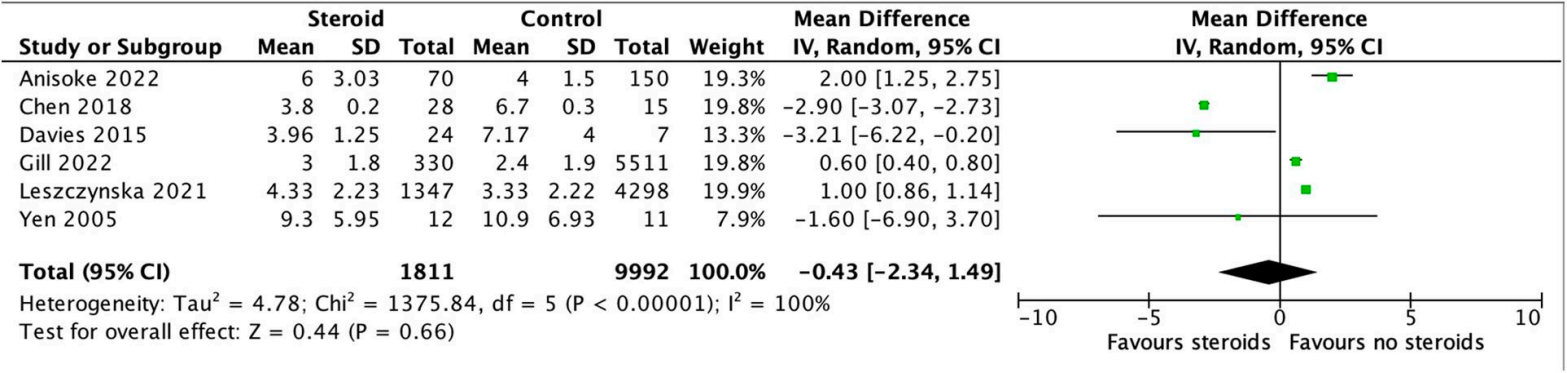
Hospital length of stay forest plot. Forest plot of mean difference in hospital length of stay between corticosteroid and control groups. Pooled mean difference in LOS between corticosteroid and control groups was −0.43 days (95% CI: −2.34 to 1.49; I^2^ = 100%; random-effects model).

The meta-analysis included six studies with a total of 1,811 patients in the corticosteroid group and 9,992 in the control group. For studies that reported LOS as median and IQR, we estimated the mean and SD using validated statistical methods. The pooled analysis demonstrated no statistically significant difference in mean hospital length of stay between the corticosteroid and control groups (mean difference −0.43 days; 95% CI: −2.34 to 1.49; *p* = 0.66) (Figure 2). There was considerable heterogeneity across studies (I^2^ = 100%, *p* < 0.00001), suggesting substantial variability in study populations, corticosteroid regimens, and timing of administration, which may limit the reliability of the pooled estimate. The certainty of evidence was rated as low, downgraded due to inconsistency and imprecision (Table 2).

**Table 2:** Summary of findings.

Outcome	Studies (*n*)	Participants (steroid/control)	Effect estimate (95% CI)	Certainty of evidence (GRADE)
Hospital length of stay	6	1,811/9,992	Mean difference −0.43 days (–2.34 to 1.49)	Low
Surgical intervention	6	1,811/9,992	RR 2.08 (1.47 to 2.95)	Low
PICU admission	2	1,677/9,809	RR 1.79 (1.34 to 2.37)	Low
30-day readmission	4	1,771/9,966	RR 2.53 (1.94 to 3.29)	Moderate^1^

1 Upgraded to moderate certainty due to large, consistent effect size (RR > 2 and I^2^ = 0%) despite risk of residual confounding from unadjusted data.

Sensitivity analysis, limited to the four studies that reported LOS directly as mean and SD without requiring conversion, showed a similar direction of effect but a larger pooled mean difference (mean difference −1.68 days; 95% CI: −4.39 to 1.03; *p* = 0.22) and persistent high heterogeneity (I^2^ = 100%, *p* < 0.00001) (Supplementary Figure S1). This suggests that the inclusion of studies with estimated means and SDs did not significantly alter the overall findings.

#### 3.4.2 Surgical intervention

**Figure 3:**
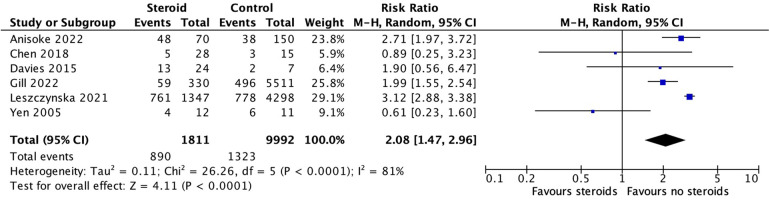
Surgical intervention forest plot. Forest plot of risk ratios for surgical intervention in corticosteroid vs. control groups. Pooled risk ratio for surgical intervention was 2.08 (95% CI: 1.47–2.96; I^2^ = 81%; random-effects model).

All six studies contributed data to this analysis, comprising 1,811 individuals in the corticosteroid group and 9,992 in the control group. Overall, corticosteroid use was associated with a significantly increased risk of surgical intervention (RR 2.08; 95% CI: 1.47–2.96; *p* < 0.0001) (Figure 3). Most studies did not distinguish surgical timing; however, Leszczyńska et al. stratified procedures by whether they occurred within the first two days of hospitalization or later (1). When early surgical cases from Leszczyńska et al. were excluded from the meta-analysis, the association between corticosteroid use and surgical risk was no longer statistically significant (RR 1.51; 95% CI: 0.97–2.33; *p* = 0.07) (Supplementary Figure S2). Significant heterogeneity was observed in both analyses (I^2^ = 80%, *p* < 0.0001), indicating variability across studies, which may reduce confidence in the pooled estimate. The certainty of this evidence was rated as low due to substantial heterogeneity and risk of bias from unadjusted confounding (Table 2).

#### 3.4.3 PICU admission

**Figure 4:**
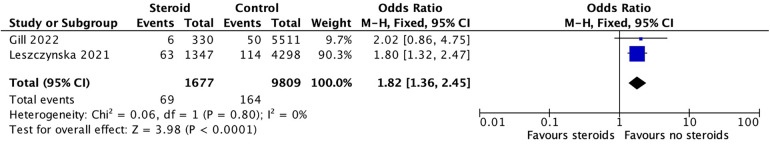
PICU admission forest plot. Forest plot of risk ratios for PICU admission in corticosteroid vs. control groups. Pooled risk ratio for PICU admission was 1.82 (95% CI: 1.36–2.45; I^2^ = 0%; fixed-effects model).

The meta-analysis included two studies comprising 1,677 patients in the corticosteroid group and 9,809 in the control group. The pooled results indicated a significantly higher risk of PICU admission among patients who received corticosteroids compared to those who did not (RR 1.82; 95% CI: 1.36–2.45; *p* < 0.0001). Heterogeneity was low (I^2^ = 0%, *p* = 0.80), supporting consistency of the observed effect across studies (Figure 4). While Gill et al. (5) did not report the timing of PICU admissions, Leszczyńska et al. stratified admissions by whether they occurred within the first two days of hospitalization or later (1, 5). When early PICU admissions from Leszczyńska et al. were excluded from the meta-analysis, the association between corticosteroid use and PICU admission remained statistically significant (RR 2.30; 95% CI: 1.13–4.71; *p* = 0.02) (Supplementary Figure S3). Despite the consistent effect, the certainty of evidence was rated as low due to risk of confounding and lack of adjustment for illness severity (Table 2).

#### 3.4.4 30-day readmission

**Figure 5:**
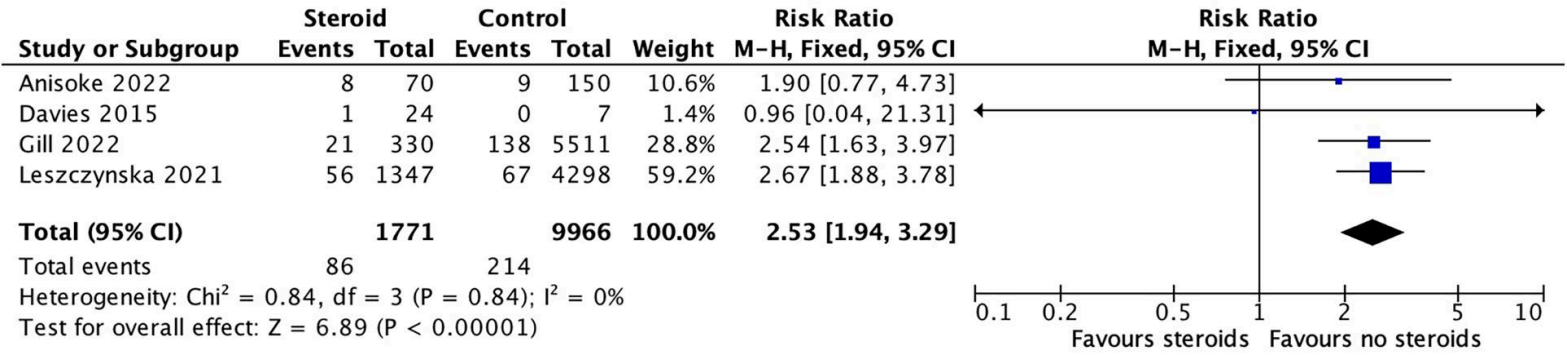
30-day readmission forest plot. Forest plot of risk ratios for 30-day hospital readmission in corticosteroid vs. control groups. Pooled risk ratio for 30-day hospital readmission was 2.53 (95% CI: 1.94–3.29; I^2^ = 0%; fixed-effects model).

The meta-analysis included four studies with 1,771 individuals in the steroid group and 9,966 individuals in the control group. The pooled analysis demonstrated a significantly higher risk of readmission among patients who received corticosteroids (RR 2.53; 95% CI: 1.94–3.29; *p* < 0.00001). Heterogeneity was low (I^2^ = 0%, *p* = 0.84), indicating consistent findings across studies (Figure 5). This outcome was rated as moderate certainty due to the large and consistent effect size despite potential residual confounding (Table 2).

#### 3.4.5 Publication bias

For hospital length of stay, funnel plot inspection demonstrated minimal visual asymmetry. In contrast, funnel plots for surgical intervention and 30-day readmission suggested possible asymmetry, with smaller studies tending to report larger effect sizes (Supplementary Figure S4). However, interpretation of funnel plots is limited by the small number of included studies and reliance on visual inspection, and these findings should therefore be interpreted with caution.

## 4 Discussion

This study provides a comprehensive synthesis of pediatric-specific evidence evaluating the use of systemic corticosteroids in orbital cellulitis. Although corticosteroids are commonly used for their anti-inflammatory effects, our findings do not demonstrate a clear clinical benefit and instead suggest possible associations with worse outcomes that should be interpreted with caution.

We found no statistically or clinically meaningful reduction in hospital length of stay. Although mean stay was slightly shorter in the corticosteroid group, this difference was not statistically significant. Importantly, the substantial heterogeneity across studies limits the reliability of this finding. This variability likely reflects differences in corticosteroid timing, dosing, and indications for initiation. For example, most studies evaluated steroids as part of initial management, whereas Davies et al. initiated treatment after clinical improvement, limiting comparability (4). Additionally, Gill et al. defined controls based on the absence of steroid use on hospital day 0, potentially misclassifying patients who received steroids later in their course (5). These differences underscore the challenges of drawing firm conclusions from retrospective data.

Corticosteroid use was associated with increased risks of surgical intervention, PICU admission, and 30-day readmission. However, these associations are likely influenced by confounding by indication, as children with more severe disease may have been more likely to receive corticosteroids and were also at higher baseline risk for adverse outcomes. This is supported by attenuation of the surgical association after exclusion of early procedures. In contrast, the association with PICU admission persisted even after excluding early admissions and was consistent across studies, suggesting that this finding may not be fully explained by baseline severity alone, although residual confounding remains likely.

Corticosteroid use was also consistently associated with higher rates of 30-day readmission. Three of four studies, including the two largest cohorts, demonstrated this association, while the smaller study by Davies et al. was underpowered and did not materially influence the pooled estimate. One possible explanation is that corticosteroid-treated patients may experience transient clinical improvement, leading to earlier discharge before complete resolution of infection. This may result in rebound inflammation or incomplete recovery, although differences in underlying disease severity or treatment response cannot be excluded.

The biological rationale for corticosteroid use is based on their ability to reduce inflammation, edema, and intra-orbital pressure, which could theoretically accelerate recovery. However, corticosteroids may also suppress immune responses, mask clinical signs of disease progression, and complicate assessment of treatment response (18). These effects may contribute to premature discharge, delayed recognition of worsening infection, or progression to more severe disease requiring intensive care. Although these mechanisms are biologically plausible, they remain hypothetical and were not directly evaluated in the included studies. Steroid-induced leukocytosis and hyperglycemia, though not reported in the included studies, are additional considerations that may complicate infection management (19). Similar risks have been described in other pediatric infections, such as pneumonia and meningitis, further supporting caution (20, 21).

Taken together, these findings suggest that routine use of corticosteroids in pediatric orbital cellulitis should be approached with caution. Current pediatric and infectious disease guidelines do not provide clear recommendations for corticosteroid use in this condition, and our results support a conservative approach (6). Until high-quality randomized trials are available, corticosteroids should be reserved for carefully selected patients in whom inflammation clearly predominates, with close clinical monitoring.

All included studies were conducted in the United States, where access to imaging, surgical drainage, and intensive care is relatively high. In low- and middle-income countries, where the burden of pediatric orbital cellulitis may be greater and access to advanced care more limited, the risks of corticosteroid use could be magnified. Moreover, no study stratified outcomes by race, ethnicity, or socioeconomic status, leaving uncertainty about whether corticosteroid-associated harms disproportionately affect vulnerable populations. Thus, risks observed in well-resourced U.S. settings may underestimate those in other contexts.

Our findings are broadly consistent with larger multicenter observational studies that have not demonstrated clear benefit and have raised concerns about potential associations with adverse outcomes (1, 5). In contrast, earlier smaller studies suggested faster recovery with corticosteroid use (2–4), highlighting the variability in existing evidence and the influence of study design and sample size.

This study has several important limitations. All included studies were observational, and pooled analyses were based on unadjusted data, limiting the ability to account for confounding factors. In particular, most studies did not adequately control for disease severity, which is likely a key determinant of both corticosteroid use and clinical outcomes. Additionally, heterogeneity in corticosteroid regimens, timing of administration, and outcome definitions may have influenced results. Although funnel plot analyses suggested possible asymmetry for some outcomes, the small number of studies limits the reliability of these assessments.

Future research should prioritize prospective, pediatric-specific randomized controlled trials with standardized corticosteroid protocols and validated measures of disease severity to better define the role of corticosteroids in orbital cellulitis.

## 5 Conclusion

Adjunctive systemic corticosteroids were not associated with a reduction in hospital length of stay in children with orbital cellulitis and may be associated with higher risks of surgical intervention, PICU admission, and 30-day readmission. However, these findings are based primarily on observational data and are likely influenced by confounding by indication and lack of adjustment for disease severity. Current evidence does not support routine use of corticosteroids in pediatric orbital cellulitis. High-quality, pediatric-specific randomized controlled trials are needed to better define their role in clinical management.

## Data Availability

The original contributions presented in the study are included in the article/Supplementary Material, further inquiries can be directed to the corresponding author.

## References

[1] Leszczynska MA Sochet AA Nguyen ATH Mateus J Morrison JM. Corticosteroids for acute orbital cellulitis. Pediatrics. (2021) 148(5):e2021050677. 10.1542/peds.2021-050677 34697218

[2] Brameli A Ashkenazi-Hoffnung L Giloni D Friling R Chodick G Landau D Systemic corticosteroids may be beneficial for managing severe or refractory orbital cellulitis in children. Acta Paediatr. (2018) 107(11):2028–9. 10.1111/apa.14467 29920768

[3] Chen L Silverman N Wu A Shinder R. Intravenous steroids with antibiotics on admission for children with orbital cellulitis. Ophthalmic Plast Reconstr Surg. (2018) 34(3):205–8. 10.1097/IOP.0000000000000910 28369021

[4] Davies BW Smith JM Hink EM Bansal R Durairaj VD. C-reactive protein as a marker for initiating steroid treatment in children with orbital cellulitis. Ophthalmic Plast Reconstr Surg. (2015) 31(4):286–9. 10.1097/IOP.0000000000000224 25393908

[5] Gill PJ Mahant S Hall M Parkin PC Shah SS Wolter NE Association between corticosteroids and outcomes in children hospitalized with orbital cellulitis. Hosp Pediatr. (2022) 12(1):70–89. 10.1542/hpeds.2021-005910 34877598

[6] Kornelsen E Mahant S Parkin P Ren LY Reginald YA Shah SS Corticosteroids for periorbital and orbital cellulitis. Cochrane Database Syst Rev. (2021) 4(4):CD013535. 10.1002/14651858.CD013535.pub2 33908631 PMC8092453

[7] Pushker N Bajaj MS Chandra M. Role of systemic corticosteroids in the management of orbital cellulitis. Ophthalmology. (2013) 120(1):193–4. 10.1016/j.ophtha.2012.10.003 23622565

[8] Higgins JPT Thomas J Chandler J Cumpston M Li T Page MJ Cochrane Handbook for Systematic Reviews of Interventions. Version 6.5. London: Cochrane (2024). Available online at: https://training.cochrane.org/handbook (Accessed May 20, 2025).

[9] Page MJ McKenzie JE Bossuyt PM Boutron I Hoffman TC Mulrow CD The PRISMA 2020 statement: an updated guideline for reporting systematic reviews. Br Med J. (2021) 372:n71. 10.1136/bmj.n71 33782057 PMC8005924

[10] EndNote. EndNote—The Best Citation & Reference Management Tool. Philadelphia (PA): Clarivate (2025). Available online at: https://endnote.com (Accessed 28 May, 2025).

[11] Ouzzani M Hammady H Fedorowicz Z Elmagarmid A. Rayyan—a web and mobile app for systematic reviews. Syst Rev. (2016) 5(1):210. 10.1186/s13643-016-0384-4 27919275 PMC5139140

[12] Wells GA Shea B O'Connell D Peterson J Welch V Losos M The Newcastle-Ottawa Scale (NOS) for Assessing the Quality of Nonrandomised Studies in Meta-Analyses. Ottawa: Ottawa Hospital Research Institute (2011). Available online at: https://www.ohri.ca/programs/clinical_epidemiology/oxford.asp (Accessed 28 May, 2025).

[13] Cochrane. Core Software for Cochrane Reviews. London: Cochrane. Available online at: https://training.cochrane.org/online-learning/core-software (Accessed 15 July, 2025).

[14] Abbas A Hefnawy MT Negida A. Meta-analysis accelerator: a comprehensive tool for statistical data conversion in systematic reviews with meta-analysis. BMC Med Res Methodol. (2024) 24:243. 10.1186/s12874-024-02356-6 39425031 PMC11487830

[15] Wan X Wang W Liu J Tong T. Estimating the sample mean and standard deviation from the sample size, median, range and/or interquartile range. BMC Med Res Methodol. (2014) 14:135. 10.1186/1471-2288-14-135 25524443 PMC4383202

[16] Anosike BI Ganapathy V Nakamura MM. Epidemiology and management of orbital cellulitis in children. J Pediatric Infect Dis Soc. (2022) 11(5):214–20. 10.1093/jpids/piac006 35438766 PMC9155619

[17] Yen MT Yen KG. Effect of corticosteroids in the acute management of pediatric orbital cellulitis with subperiosteal abscess. Ophthalmic Plast Reconstr Surg. (2005) 21(5):363–6. 10.1097/01.iop.0000176536.73642.2c 16234700

[18] Cain DW Cidlowski JA. Immune regulation by glucocorticoids. Nat Rev Immunol. (2017) 17(4):233–47. 10.1038/nri.2017.1 28192415 PMC9761406

[19] Chaudhuri D Israelian L Putowski Z Prakash J Pitre T Nei AM Adverse effects related to corticosteroid use in sepsis, acute respiratory distress syndrome, and community-acquired pneumonia: a systematic review and meta-analysis. Crit Care Explor. (2024) 6(4):e1071. 10.1097/CCE.0000000000001071 38567382 PMC10986917

[20] Stern A Skalsky K Avni T Carrara E Leibovici L Paul M. Corticosteroids for pneumonia. Cochrane Database Syst Rev. (2017) 12(12):CD007720. 10.1002/14651858.CD007720.pub3 29236286 PMC6486210

[21] Brouwer MC McIntyre P Prasad K van de Beek D. Corticosteroids for acute bacterial meningitis. Cochrane Database Syst Rev. (2015) 2015(9):CD004405. 10.1002/14651858.CD004405.pub5 26362566 PMC6491272

[22] Egger M Smith GD Schneider M Minder C. Bias in meta-analysis detected by a simple, graphical test. Br Med J. (1997) 315(7109):629–34. 10.1136/bmj.315.7109.629 9310563 PMC2127453

[23] Schünemann HJ Brożek J Guyatt GH Oxman AD editors. GRADE Handbook. Hamilton: The GRADE Working Group (2013). Available online at: https://gdt.gradepro.org/app/handbook/handbook.html (Accessed June 6, 2025).

