# Supplementary material for: Adjunctive systemic corticosteroids in pediatric orbital cellulitis: a systematic review and meta-analysis
Source: Front Pediatr. 2026 Apr 20;14:1794826. doi: 10.3389/fped.2026.1794826 (PMC13136254; doi:10.3389/fped.2026.1794826)
Supplement: Supplementary file 1 [file Table1.docx]

**Supplementary Table S1. PRISMA Checklist**

| Section/Topic | Item # | Checklist Item | Location in Manuscript |
| --- | --- | --- | --- |
| Title | 1 | Identify the report as a systematic review/meta-analysis | Title |
| Abstract | 2 | Provide a structured summary | Abstract |
| Introduction | 3 | Describe rationale for the review | Introduction |
| Introduction | 4 | Provide explicit statement of objectives | Introduction |
| Methods | 5 | Eligibility criteria | Methods – Eligibility criteria |
| Methods | 6 | Information sources | Methods – Search strategy |
| Methods | 7 | Search strategy | Methods – Search strategy |
| Methods | 8 | Selection process | Methods – Study selection |
| Methods | 9 | Data collection process | Methods – Data extraction |
| Methods | 10 | Data items | Methods – Data extraction |
| Methods | 11 | Study risk of bias assessment | Methods – Quality assessment |
| Methods | 12 | Effect measures | Methods – Statistical analysis |
| Methods | 13 | Synthesis methods | Methods – Statistical analysis |
| Methods | 14 | Reporting bias assessment | Methods – Statistical analysis |
| Methods | 15 | Certainty assessment | Methods – Statistical analysis |
| Results | 16 | Study selection | Results – Database search |
| Results | 17 | Study characteristics | Results – Baseline characteristics |
| Results | 18 | Risk of bias in studies | Results – Quality assessment |
| Results | 19 | Results of individual studies | Results – Outcomes |
| Results | 20 | Results of syntheses | Results – Outcomes |
| Results | 21 | Reporting biases | Results – Outcomes, Publication bias |
| Results | 22 | Certainty of evidence | Results – Outcomes |
| Discussion | 23 | General interpretation of results | Discussion |
| Discussion | 24 | Limitations of evidence | Discussion |
| Discussion | 25 | Implications for practice/research | Discussion |
| Other | 26 | Registration and protocol | Methods – Protocol and registration |
| Other | 27 | Support, competing interests, data availability | Disclosure |
