# Supplementary material for: Adjunctive systemic corticosteroids in pediatric orbital cellulitis: a systematic review and meta-analysis
Source: Front Pediatr. 2026 Apr 20;14:1794826. doi: 10.3389/fped.2026.1794826 (PMC13136254; doi:10.3389/fped.2026.1794826)
Supplement: Supplementary file 2 [file Datasheet1.docx]

**Supplemental Appendix 1. Search Strategy**

A comprehensive literature search was conducted in PubMed, Embase, and the Cochrane Library from database inception through June 1^st^, 2025. The full search strategies for each database are provided below.

**PubMed**

("Orbital cellulitis"[MeSH Terms] OR "orbital cellulitis"[Title/Abstract])
AND
("Corticosteroids"[MeSH Terms] OR "corticosteroids"[Title/Abstract] OR "steroids"[Title/Abstract] OR "glucocorticoids"[Title/Abstract])
AND
("Pediatrics"[MeSH Terms] OR "Child"[MeSH Terms] OR "Adolescent"[MeSH Terms] OR child*[Title/Abstract] OR pediatric*[Title/Abstract])
AND
(Humans[MeSH Terms])
AND
(English[lang])

**Cochrane Library**

("orbital cellulitis")
AND
(corticosteroids OR steroids OR glucocorticoids)
AND
(child OR children OR pediatric OR adolescent)

**Embase**

('orbital cellulitis'/exp OR 'orbital cellulitis':ti,ab)
AND
('corticosteroid'/exp OR 'corticosteroids':ti,ab OR 'steroids':ti,ab OR 'glucocorticoids':ti,ab)
AND
('child'/exp OR 'pediatrics'/exp OR 'adolescent'/exp OR child*:ti,ab OR pediatric*:ti,ab OR adolescent*:ti,ab)
AND
[humans]/lim
AND
[english]/lim
