# Supplementary material for: Adjunctive systemic corticosteroids in pediatric orbital cellulitis: a systematic review and meta-analysis
Source: Front Pediatr. 2026 Apr 20;14:1794826. doi: 10.3389/fped.2026.1794826 (PMC13136254; doi:10.3389/fped.2026.1794826)
Supplement: Supplementary file 3 [file Supplementaryfile1.docx]

**
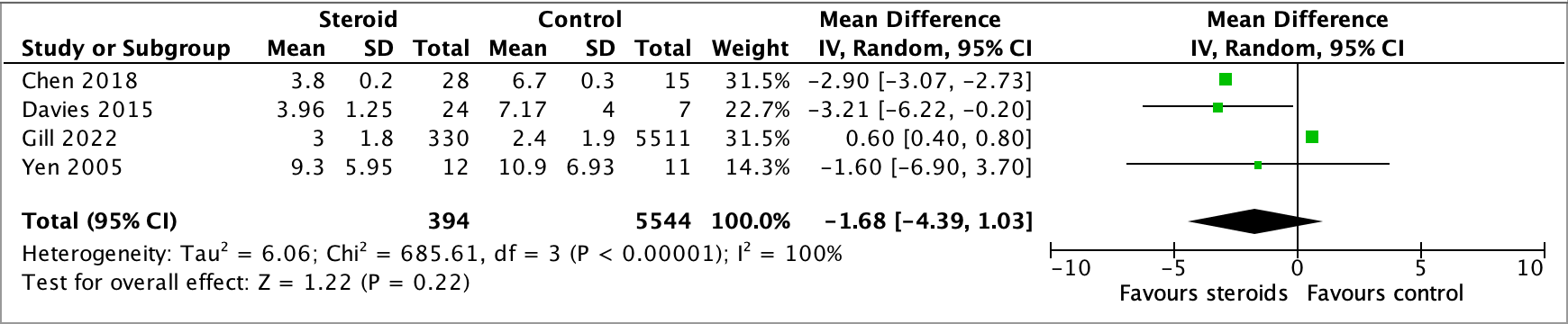
**

**Supplemental Figure S1. Hospital LOS Sensitivity Analysis.** Forest plot including only studies that directly reported mean and standard deviation. Pooled mean difference –1.68 days (95% CI –4.39 to 1.03; I² = 100%; random-effects model).
