# Supplementary material for: Adjunctive systemic corticosteroids in pediatric orbital cellulitis: a systematic review and meta-analysis
Source: Front Pediatr. 2026 Apr 20;14:1794826. doi: 10.3389/fped.2026.1794826 (PMC13136254; doi:10.3389/fped.2026.1794826)
Supplement: Supplementary file 4 [file Supplementaryfile2.docx]

**
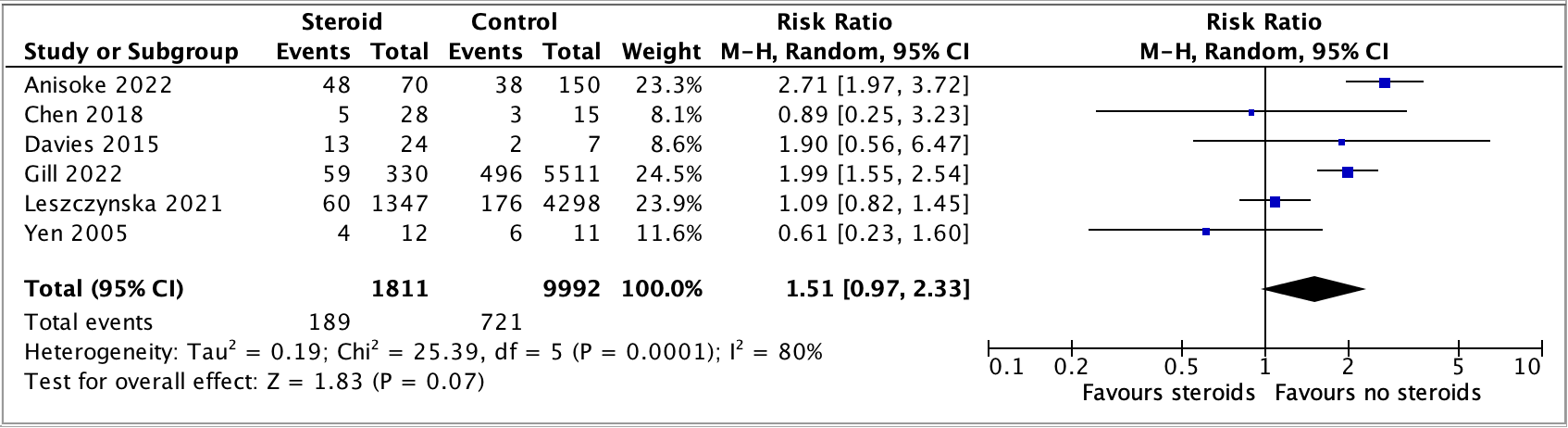
**

**Supplemental Figure S2. Late Surgical Intervention Forest Plot.** Forest plot excluding procedures performed within the first two days of admission. Association between corticosteroid use and surgery was not statistically significant (RR 1.51, 95% CI 0.97–2.33; I² = 80%; random-effects model).
