# Supplementary material for: Adjunctive systemic corticosteroids in pediatric orbital cellulitis: a systematic review and meta-analysis
Source: Front Pediatr. 2026 Apr 20;14:1794826. doi: 10.3389/fped.2026.1794826 (PMC13136254; doi:10.3389/fped.2026.1794826)
Supplement: Supplementary file 5 [file Supplementaryfile3.docx]

**
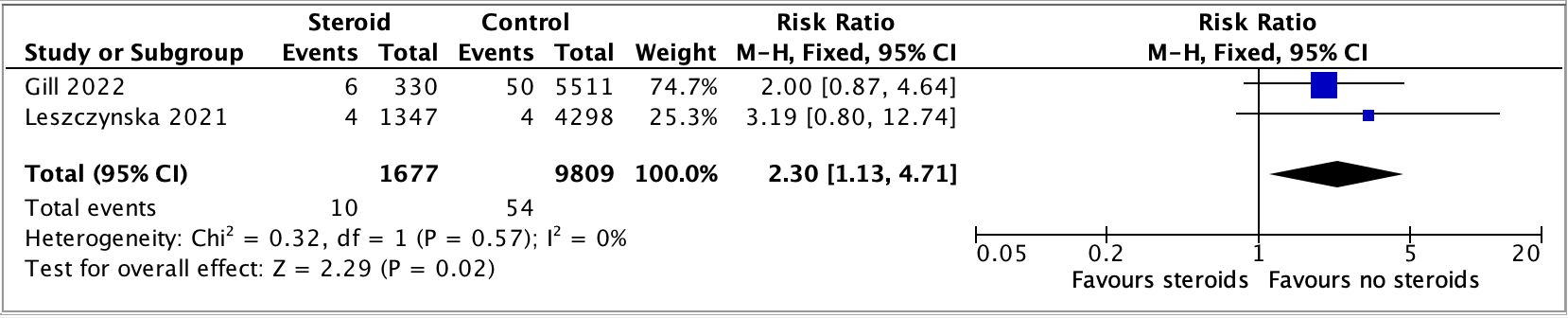
**

**Supplemental Figure S3. Late PICU Admission Forest Plot.** Forest plot excluding PICU admissions occurring within the first two days of hospitalization. Corticosteroid use remained associated with higher risk of PICU admission (RR 2.30, 95% CI 1.13–4.71; I² = 0%; fixed-effects model).
