# Supplementary material for: Adjunctive systemic corticosteroids in pediatric orbital cellulitis: a systematic review and meta-analysis
Source: Front Pediatr. 2026 Apr 20;14:1794826. doi: 10.3389/fped.2026.1794826 (PMC13136254; doi:10.3389/fped.2026.1794826)
Supplement: Supplementary file 6 [file Supplementaryfile4.docx]

**a)**

**
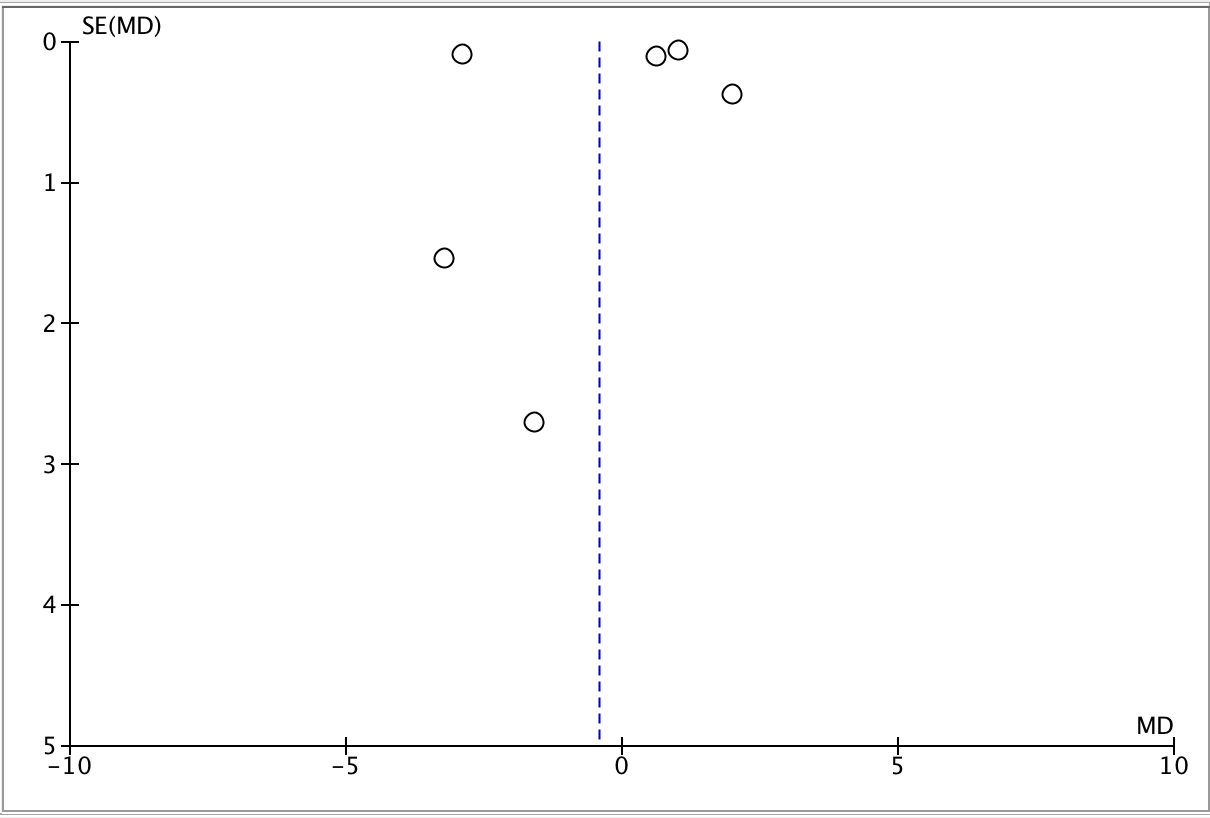
**

**b)**

**
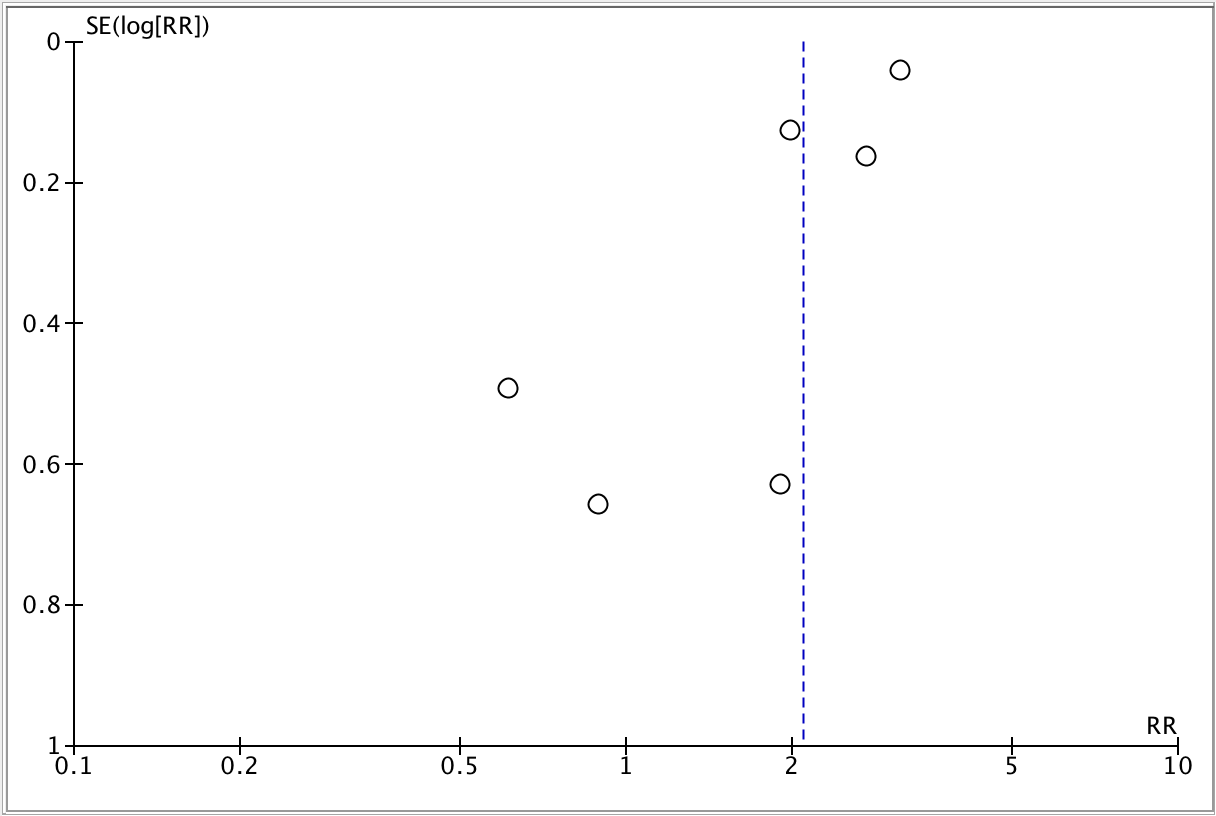
**

**c)**

**
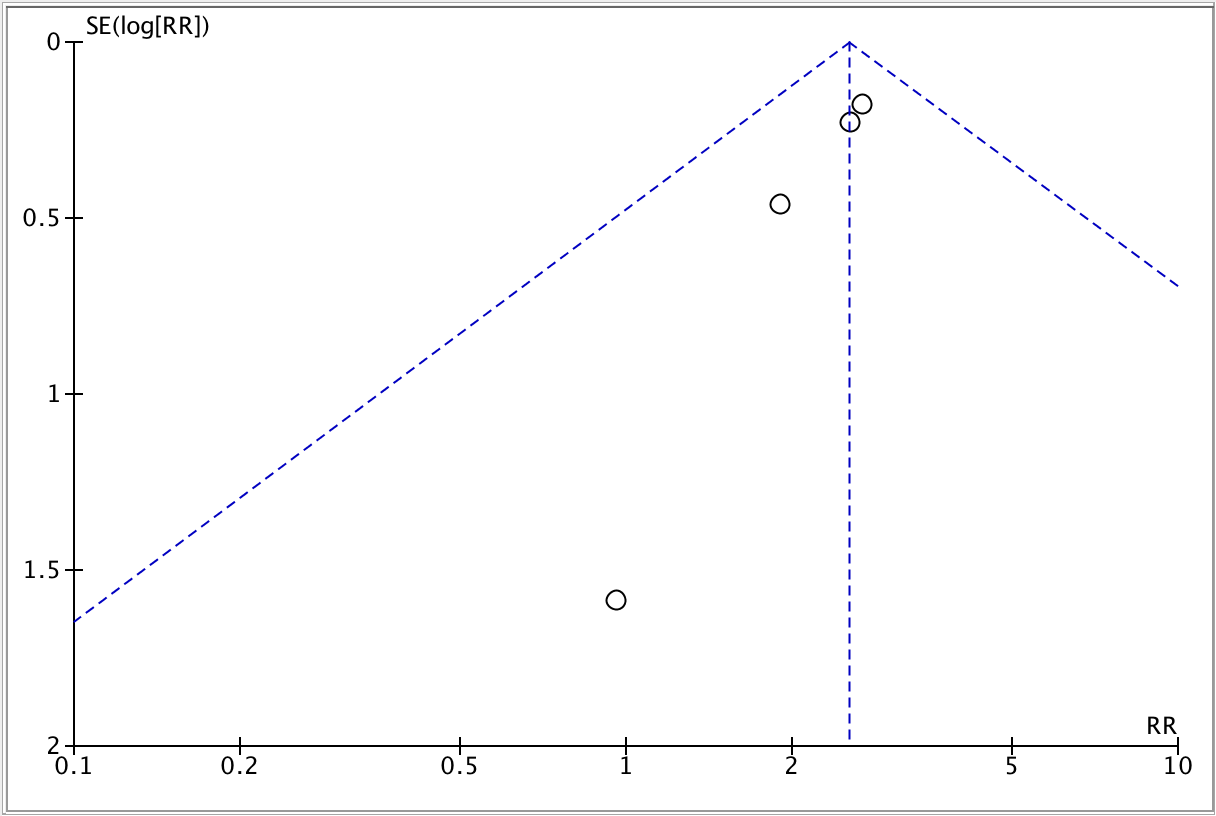
**

**Supplemental Figure S4. Funnel Plots.** a) Hospital length of stay; b) surgical intervention; c) 30-day readmission
